# Users’ involvement in mental health services: programme logic model of an innovative initiative in integrated care

**DOI:** 10.1186/s13033-016-0111-5

**Published:** 2017-01-05

**Authors:** Carolane Tremblay, Valérie Coulombe, Catherine Briand

**Affiliations:** 1 Centre for Studies on Rehabilitation, Recovery and Social Inclusion, Centre de recherche de l’Institut universitaire en santé mentale de Montréal, Montréal, QC Canada; 2 Occupational Therapy Program, School of Rehabilitation, Université de Montréal, Montréal, QC Canada; 3 Canadian Mental Health Association-Montreal Branch, Montréal, QC Canada

**Keywords:** Mental health services, Shared values, Integrated care, Partnership, Users’ involvement, Carers’ involvement, Programme logic model

## Abstract

**Background:**

Collaboration and partnership are key issues for modern health systems seeking to implement quality integrated care that meets the needs of the population. The *Carrefour Communautaire*-*Institutionnel*-*Usagers* (Connecting Community organisations-Institutions-Users, CCIU), involving community- and institution-based mental health workers, carers and users, is an innovative normative integrated care group (group for shared values, culture and vision) established by the Canadian Mental Health Association-Montreal Branch. A programme evaluation approach was used to conduct a logic analysis of the CCIU in order to understand the relationships between its resources, activities and outcomes, build a common understanding and, allow for its replication.

**Methods:**

Five steps were involved in the creation of a programme logic model. A non-exhaustive literature search for similar initiatives, a review of documents related to the CCIU process and direct observations led to the development of a first model. Then, following a participatory and reflexive process, this model was validated with CCIU participants.

**Results:**

A comprehensive model and a simplified model were created. Participants’ experiential knowledge and scientific knowledge helped to identify the essential components of the successful operation of the CCIU.

**Conclusions:**

The CCIU, with its eight essential components, including relations based on equality and mutual respect, corresponds to an essential step in normative integration and integrated care that lead to improved quality services.

## Background

Collaboration and partnership are key issues for modern health systems seeking to implement quality integrated care that meets the needs of the population [1, 2]. The concept of Integrated Care is defined by Kodner and Spreeuwenberg [3, p. 3] as a practice that aims.

“To create connectivity, alignment and collaboration between the cure and the care sectors […] to enhance quality of life, consumer satisfaction and system efficiency for patients with complex, long term problems cutting across multiple services, providers and settings”.

It involves notions of patient-centred care, inter-professional working, shared decision-making, integrated delivery networks, and different levels of integration (clinical, organisational, normative, etc.) [2, 4].

In the area of mental health where users have complex and long-term problems, several initiatives (stemming from policies, demands, etc.) support an integrated—and collaborative—care practice and promote the active involvement of users in the development and organisation of services [5–12]. In several countries, including Canada, the United States, Great Britain and Australia, the need to involve users and their carers in the reform of mental health services and the implementation of integrated care is now widely endorsed [11, 13–20]. In particular, in Canada (including the province of Quebec), government policies have identified, among their priorities and strategic directions, expanding the role of users and all partners in the various decision-making levels and structures [16, 21].

However, this new way of thinking and organising services gives rise to numerous challenges, in particular regarding the notion of normative integration (shared values, culture and vision) [2]. The different philosophies and ideologies guiding stakeholders make collaborative partnerships difficult. The challenging partnership between users and other stakeholders in the system as well as between the stakeholders within the same local network (in particular, between community and institutional stakeholders) remains a highly prevalent issue [7, 22]. Stakeholders within the same mental health services network have difficulty working together. Despite political will and recommendations in favour of networking and integrated care [17, 22–24], stakeholders have little space for discussing shared issues (including integrated care) and building a common vision of services. Moreover, they have even fewer means to really involve users and carers in the planning and organisation of services.

Thus, in Canada, the Canadian Mental Health Association-Montreal Branch (CMHA-Montreal), a community organisation dedicated to mental health promotion and prevention of mental illness, has set up an innovative initiative called the *Carrefour Communautaire*-*Institutionnel*-*Usagers* (Connecting Community organisations-Institutions-Users, hereafter the CCIU) (formerly called *Carrefour Communautaire*-*Institutionnel,* Connecting Community organisations-Institutions, the CCI) [25]. The CCIU is mandated to promote the participation of users and carers in the planning, organisation and evaluation of mental health services in order to improve services and coordinate care by providing a forum where ideas can be exchanged and freely expressed [26]. The main goals of the CCIU involve: (1) forging connections and sharing viewpoints among the stakeholders in order to develop better knowledge and a better understanding of lived experience, in particular that of users and carers (2) sharing information regarding the settings and organisations that the participants are involved in, and (3) promoting users’ involvement in the organisation of services and better services by considering the advantages of integrated care (in particular the notion of normative and horizontal integration) [2, 27].

The CCI emerged in 1998 out of a will to forge connections between mental health workers from community organisations and institutional settings. However, over time, it became necessary to involve users and carers in the dialogues. In fact, users’ involvement is, in our view, one of the means—certainly an essential step—to openly support the notion of integrated care. Thus, in 2009, the CCIU was created, formalising the presence of these four types of actors (community and institutional stakeholders, users, carers) and enabling them to exchange ideas on various topics related to mental health (between 15 and 20 participants per meeting). Participation is voluntary with great concern for a representative group of all actors. Importance was given to having an equal number of participants from diverse backgrounds in the forum: a minimum of 50% of users and carers is thus required. To date, the CCIU has been evaluated through internal survey only. It has never been systematically evaluated based on a logic analysis of its components so as to gain a more accurate understanding of how it operates.

### Purpose

This article aims to present the results of the logic analysis of the CCIU, undertaken in order to understand how this initiative operates, i.e. the relationships that exist between its resources, activities and short-term, intermediate and long-term outcomes. Programme logic analysis is a component of evaluative research that assesses the relationship between the different components of the intervention implemented [28, 29]. Modelling is performed to identify and put forward a graphic presentation of the mechanisms of action used to achieve the programme’s goals. In this project, the CCIU is the proposed programme.

Logic analysis is an essential step to implement and replicate this same initiative elsewhere as well as the step prior to a systematic evaluation of its effects [28, 29]. Also, benefits of using the logic model tool are many for an organisation and stakeholders: (1) builds a common understanding of the programme and expectations for resources, customers reached, and results, (2) helps for sharing ideas, identifying assumptions, team building, and communication, (3) allows critical self-evaluation and identifying components that are crucial to goal attainment, inconsistent or non essential, by example through a relevant scientific literature [30]. Three approaches are described to explain the use of logic models: (1) strategic assessment approach, which is driven through on-going dialogue with programme staff and participants, (2) policy-scientific approach, which is more empirical and consists of generating assumptions that have been made about how the programme is supposed to work, and (3) elicitation method, which aims at recovering the mental models that programme staff hold about the programme.

## Methods

The logic analysis of the CCIU was conducted in five steps, as suggested by McLaughlin and Jordan [30, pp. 61–72], including, among other things, a review of the grey and scientific literature as well as direct observations and feedback from CCIU participants. These five steps are described in detail as follows:

1. *Collecting the relevant information* Important data used to describe and understand the CCIU were collected in three ways. First, the documentation produced by the CMHA-Montreal was reviewed (previous meeting reports, conference presentations, internal documents, presentation booklet, annual evaluation reports). Second, direct observations were carried out by one of the authors of this article (CT) who participated in the CCIU meetings over the course of 1 year. Third and lastly, a non-exhaustive review of the literature was conducted to determine whether or not initiatives that are similar to the CCIU existed, using *PubMed* and *PsychInfo (*selecting the keywords “user* involvement” OR “user* participation” AND “mental health services”). In order to be included in our exploratory review, the articles had to pertain to specific criteria for inclusion or exclusion (Table 1; Fig. 1).

**Table 1:** Inclusion and exclusion criteria

Inclusion criteria	Exclusion criteria
Discussions between mental health network actors on mental health-related topics	User’s participation within the context of teaching or research
Meetings outside the usual institutional structure	Participation in a group by or for users (peer support)
Sharing of experiential and theoretical knowledge	Participation in one’s own treatment

**Fig. 1:**
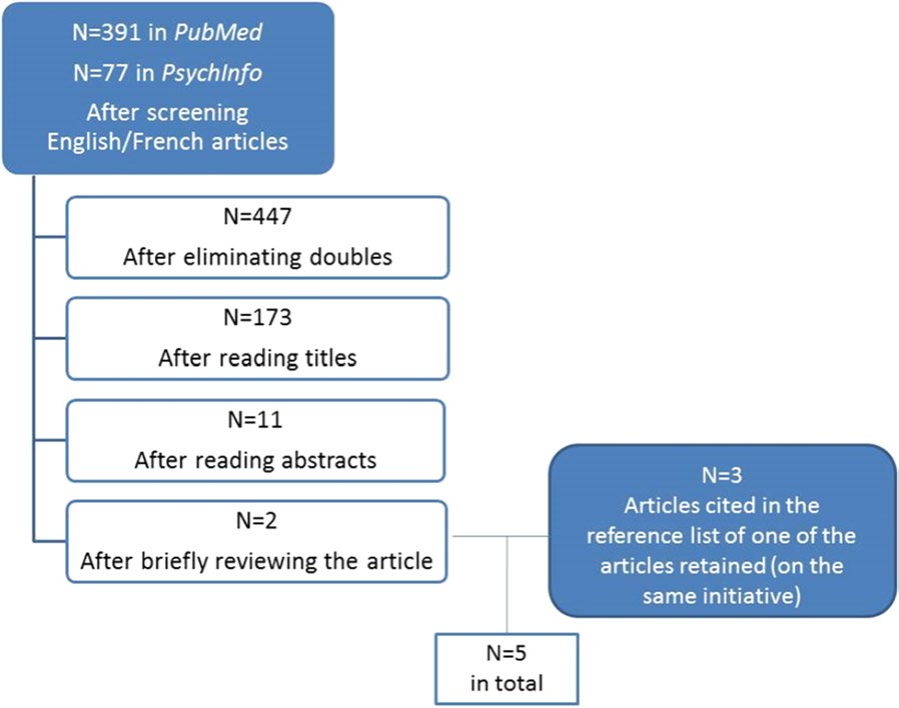
Results of non-exhaustive literature review
2. *Clearly defining the problem to be solved by the programme* The issue addressed by the CCIU and the context of its existence were defined in collaboration with the CMHA-Montreal. Thus, throughout this process, six meetings were held between the research team and the CMHA-Montreal (excluding the meetings with CCIU participants). These meetings helped to better coordinate the goals and guidelines of the project as pursued by the CMHA-Montreal and the research team, and to validate their understanding of the model, its goals and its mode of operation.
3. *Defining the elements of the model in a table* All the elements relating to the CCIU were classified in a table according to the following categories: resources, activities, outputs, short-term outcomes, intermediate outcomes, and long-term outcomes (the usual variables in a process of programme logic analysis) (Table 2).

**Table 2:** Definitions of the elements of a logic analysis

Elements	Definitions according to McLaughlin and Jordan [30, pp. 57–58]
Resources	“Human and financial resources as well as other inputs required to support the program”
Activities	“The essential action steps necessary to produce programs outputs”
Outputs	“The products, goods, and services provided to the […] program participants”
Short-term, intermediate and long-term outcomes	“Changes or benefits to people, organisations, or others program targets that are expected to result from their being exposed to activities and outputs”
4. *Developing the model* A first outline of the programme’s logic model was developed. CMapTools software (http://cmap.ihmc.us/) was used for the graphic presentation.
5. *Validating the model with stakeholders* The CCIU participants validated the model in two stages. A first validation was conducted in April 2013 with a subgroup of four participants: one institution-based mental health worker, one staff member from the CMHA-Montreal, one user and one carer (theoretical sampling). This validation led to the creation of an intermediate logic model. This version was used for a second validation with the 16 CCIU participants who were present at the meeting in May 2013 (including the CMHA-Montreal staff members), which allowed us to gain a more in-depth understanding of the programme. A comprehensive logic model and a simplified logic model resulted from this participatory construction process.

The choice of this participatory methodology was based on the CCIU’s values and principles. Participatory construction process allowed sharing ideas within the group, building a common understanding of the programme and identifying next steps for the development of the CCIU and the achievement of its objectives (strategic assessment approach, which is driven through dialogues with programme staff and participants [30]). A participatory construction process meets the concept of co-production (particularly the following principles: taking an assets-based approach and building on people’s existing capabilities) which is now current in the literature in health’s field [31]. Also, logic model allowed validating mechanisms of action with other similar initiatives and scientific literature and put forward a graphic representation of the programme (policy-scientific approach and elicitation method, which consists of generating assumptions that have been made about how the programme is supposed to work and producing comprehensive model [30]).

### Ethics, consent and permissions

Given the nature of the study (logic analysis of a program), no individual data of participants were considered. They were involved as stakeholders in building a common understanding of the programme and not as subjects of study. Despite this, all the CCIU participants, the stakeholders involved in the validation of the model and the direction of the CMHA-Montreal agreed (informal agreement) to participate in the study and publication.

## Results—description of the practice

First, the literature review helped to identify two initiatives that met the research criteria: Trialogues and Mental health forums [6, 32]. Only Trialogues turned out to be relevant to this project. A consultation of the reference lists of the articles yielded 3 additional articles relating to Trialogues [33–35]. Initiated in Germany (where they are known as Psychosis Seminars) and extended to other countries in Europe (Austria, Ireland, etc.), Trialogues allow groups of 10–60 people to exchange ideas on various topics relating to care experience, recovery, crisis management, and so on [6, 35]. Similar to the CCIU, Trialogues, established in recent years, provide a forum for dialogue between users, carers and mental health workers, outside institutional walls and on an equal footing [6, 33, 34]. However, the two initiatives appear to differ insofar as the participation of CCIU members appears to extend over a longer term [36], which could likely to result in differences regarding group dynamics and changes in perceptions and attitudes. The similarities and differences between the Trialogues and the CCIU are based on the retrieved literature review and the present study.

Second, the logic analysis helped to identify (1) the CCIU’s specific resources, activities and outputs, (2) its short-term, intermediate and long-term outcomes and, (3) the essential components that account for the achievement of its goals.

### CCIU’s resources, activities and outputs

**Fig. 2:**
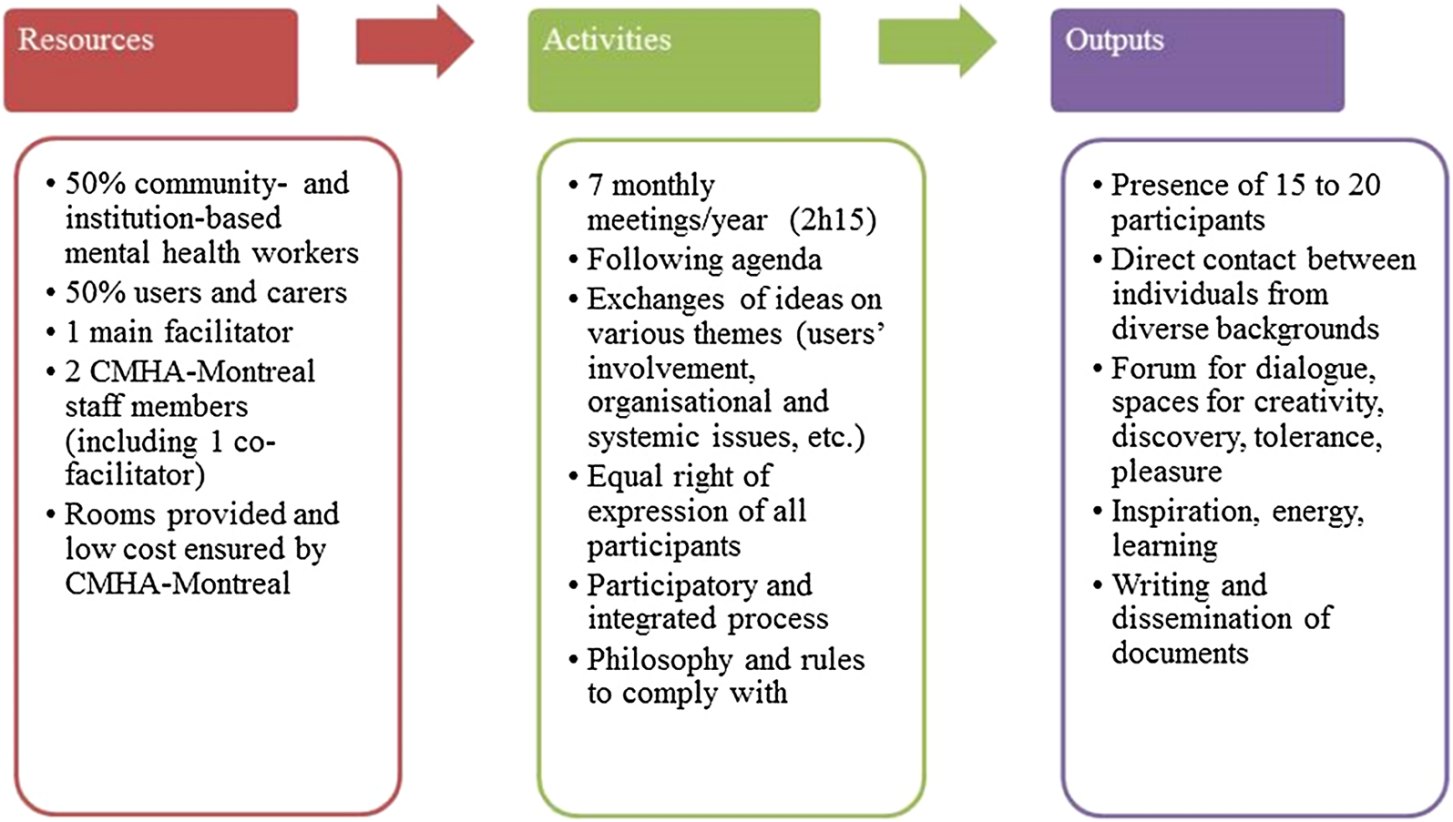
CCIU—resources, activities and outputs

The CCIU is a programme that initiates activities requiring resources and entailing outputs (see Fig. 2). At any one meeting, it calls for between 15 and 20 participants who are actively involved in the mental health network: institution-based mental health workers, mental health workers from community organisations, users and carers. Their participation outside the confines and functions of their respective organisations is voluntary and/or personal. This is meant to reduce the barriers that may be induced by the hierarchical relations that usually exist in organisations or systems. This voluntary partnership, which is intrinsically motivated, fosters collaboration and the sharing of common values and visions. Since particular attention is paid to the principle of parity (at least 50% users and carers), a balance of power and influence is aimed at. Compliance with these group norms and rules is ensured through the participation of two staff members from the CMHA-Montreal (one of whom co-facilitates the sessions). Since this organisation has credibility and is recognised for its leadership role in the mental health network, and plays no part in issues related to services offered or advocacy, it can monitor the norms and rules implemented by the CCIU in a neutral and rigorous way. It covers the low annual costs generated by the CCIU and also provides rooms for the meetings.

The CCIU holds seven monthly meetings per year, each lasting 2 h and 15 min, drawing on the knowledge of each participant. The meetings proceed according to a pre-established agenda. The CCIU’s philosophy (open-mindedness, freedom of expression, active listening, respect, and conviviality) is promoted by the main facilitator (and co-facilitator) who leads and structures the meetings. This philosophy and some others group rules (e.g., avoiding discussions on territorial and budgetary rivalries, importance attached to process rather than outcomes) foster respect for the equal right of expression of all participants and the implementation of a participatory and integrated approach. The whole group is responsible for choosing the content of the meetings, self-evaluation mechanisms used and strategy developments during the year.

Once the group dynamics have been established, these combined elements allow the participants to exchange and, share ideas and experiences on a theme. Between 15 and 20 participants attend each session (for a total of approximately 30 different participants per year) and participants can renew their participation from year to year. This direct contact with other individuals from different backgrounds who have diversified knowledge (experiential, theoretical) helps to gradually set up forums for dialogue within the group: spaces for discovery, tolerance, creation, pleasure, and so on. Each individual derives personal outputs from it, such as inspiration, energy and learning. The CMHA-Montreal is responsible for coordinating the meetings, drafting documents related to the project (reports, conference presentations) and disseminating information to ensure the follow up and continuity of the meetings.

### CCIU’s short-term, intermediate and long-term outcomes

Outcomes were identified following the logic analysis. Table 3 presents those derived from the analysis of the participants’ discourse. In the short term, the participants forged connections with one another, learned to trust each other, developed a sense of group belonging and discovered common ground. They shared their experiential knowledge, scientific knowledge and opinions through discussions on various themes (users’ involvement, available resources and services, new intervention approaches, thoughts on different issues, joint projects). Thus, they cultivated their understanding of different viewpoints. They exchanged information about events in the mental health network. Together, they explored obstacles, needs, advantages, stories of success and desired changes with regard to users’ involvement in the network and truly integrated care. As the meetings progressed, their motivation to continue participating in the meetings increased. The CCIU was an experience in itself, within which each individual developed and shared lived experience. This experience was lived differently by the participants and involved a process of working on themselves and reaching out to others.

**Table 3:** Short-term, intermediate and long-term outcomes as reported by the participants

Short-term outcomes	Intermediate outcomes	Long-term outcomes
Forging of connections and development of mutual trust Sharing of knowledge, experience, opinions Emergence of new knowledge and awareness of participants’ lived experience Enhanced knowledge of issues in the mental health network and participants’ personal, professional and organisational realities Personal growth and/or progress towards greater openness to others	Enhanced understanding of the mental health network and its issues Change in perceptions and awareness that a situation can be viewed differently Introspection and prejudice reduction Change in attitudes and empowerment Mobilisation and motivation towards change	Put person first and promote human values Change in practices and establishment of concrete projects Support for integrated practices that foster users’ active involvement Engagement and involvement in promoting recovery values

In the intermediate term, the participants developed a better and more nuanced understanding of the mental health network, complex situations and the realities experienced by the other participants, who all got involved to improve services. This may have led to greater introspection. Antagonism and prejudice between community-based mental health workers and institution-based mental health workers, users and carers were reduced. A change could be seen in their attitudes. Individuals came together and a common language emerged between them. These elements helped to develop arguments promoting an integrated approach that encourages shared philosophies and the involvement of users and carers in decision making. Several participants became agents of change by sharing their lived experience within the CCIU and what they had learned in their settings. As the group achieved a degree of maturity, it started considering actions that could be implemented to have a greater impact on the mental health network by promoting the involvement of users and carers as well as collaborative and recovery-oriented approaches to services. The group became a community-based support for collaborative action [37].

Over the long term, the individuals involved could put the person first, regardless of her roles and the settings she came from, and promote human values. Changes in the participants’ practices were envisaged, such as setting up projects aimed at eliminating gaps and at collaboration among stakeholders, participating in a societal transformation process, and promoting recovery-oriented services (recovery-oriented services promote self-management of illness and shared decision-making, recruiting peer supporters among the medical staff, advocacy and combating stigma, and increased involvement of the people concerned in decisions related to system planning [38–42]). These prospects gradually led some participants to support integrated care practices (such as joint planning, shared decisions and priorities and interorganisational working) and promote recovery values (active involvement of users and carers, etc.). There was also a desire to replicate the CCIU process in various territories. Thus, a number of initiatives were led by the participants and the CMHA-Montreal.

### CCIU’s essential components

**Fig. 3:**
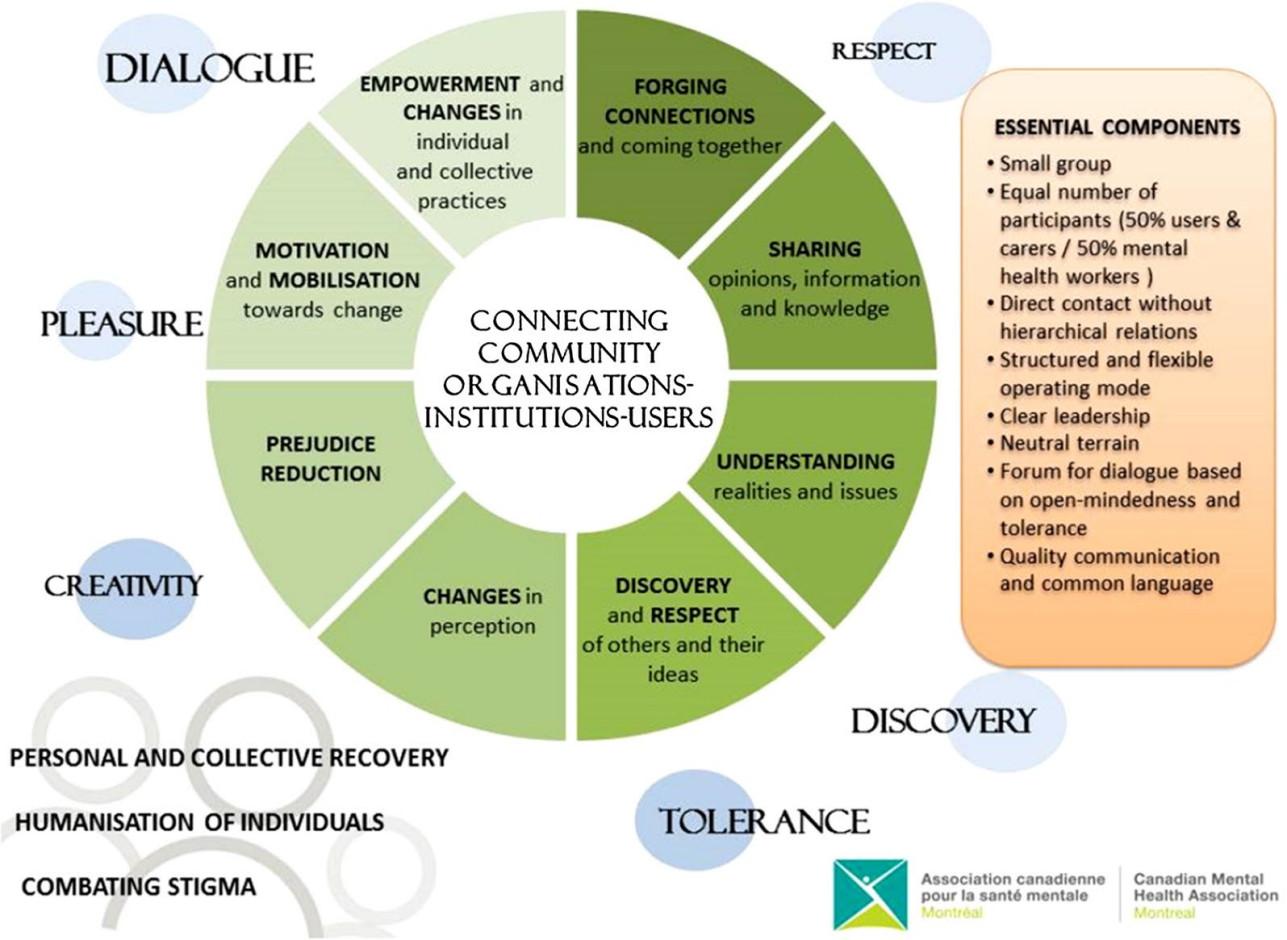
Simplified model of the CCIU

A reflexive process conducted with the participants and an analysis of notions related to group theories helped to identify eight essential components of the CCIU that contributed to the emergence of the outcomes mentioned above. These results are reflected in the simplified model (Fig. 3).

- The type of group, described as circumscribed or optimal, allows for interactions between a small number of individuals with a common target [43, 44].
- An equal number of participants from different backgrounds allows for the same amount of speaking time to be accorded to different types of knowledge and makes it easier for participants to speak up [43].
- Direct contact without hierarchical relations between the participants can lead to a change in attitudes since this strategy is conducive to prejudice reduction [45–47].
- The CCIU’s structure is based on precise group rules and a philosophy and values of open-mindedness and mutual respect. This operating mode, which is both structured and flexible, is considered to be an essential component of the success of meetings and facilitates the integration of new members with those who have been attending the meetings for a long time [44].
- The quality of the leadership of the CCIU’s facilitator and co-facilitator, as well as the coordination by CMHA-Montreal, were highlighted by the majority of participants. The leader plays a major and important role with regard to the meetings’ content, links with the participants and group dynamics [48]. Among other things, this individual facilitates the members’ participation and helps to objectify and clarify some interventions [44].
- The place where the meetings are held, which is outside the participants’ workplaces and care settings, described as neutral terrain, is crucial to the successful operation of the group. Indeed, holding meetings within the usual institutional structures or only in hospital settings would probably bring about barriers preventing the sharing of lived experience [49].
- Based on the richness of the discussions within the CCIU, it can be affirmed that the group is a forum for dialogue where open-mindedness and tolerance prevail [43], which is consistent with the way the participants described themselves (“participatory,” “tolerant” and “free”). Dialogue, considered to be a key aspect of the CCIU, allows for exchanges of ideas on an equal footing and brings about changes. For some participants, the CCIU was the only forum in which they felt able to speak freely within the mental health network and, probably, the only forum in which they could learn to express their ideas.
- The common language that is created between the participants is equally important since effective and quality communication is essential in a group process and for group cohesion [43]. The group dynamics, described as “harmonious and welcoming,” aptly illustrate the energy that exists within the CCIU [44].

## Discussion

The goals of the CCIU are to forge connections between the different stakeholders in the mental health network, allow them to exchange ideas and opinions as well as share information regarding organisational and systemic work values and philosophies in order to promote integrated care—in particular, normative integration and greater involvement of users and their carers in the organisation and planning of services [2]. The logic analysis showed that various mechanisms have been used to achieve these goals. First, by forging connections between individuals who voluntarily attended the meetings, mutual trust has gradually been built and the resulting openness to others has led to better knowledge and understanding of individual and collective realities as well as a better understanding of the frames of reference (recovery model of care, integrated care, etc.) [2]. These elements have led to changes in perceptions and attitudes and have helped to gradually reduce prejudice and encourage, among all the CCIU participants, personal introspection and shared values and philosophies that have led to mobilisation towards changes in practice. Since the CCIU—in its current form involving 50% users and carers—is a relatively recent initiative (2009), it is difficult to demonstrate and evaluate the concrete changes in practice and the actual projects engaged in by the participants outside the CCIU. Nevertheless, several participants consulted for this logic analysis agreed that the CCIU has enabled them to gradually progress towards these changes in practice. The CCIU also further help participants to implement concrete projects to reduce the gaps between the values of community organisations and institutions as well as between service providers and users. Moreover, the CCIU has allowed them to gradually get involved in collaborative and recovery-oriented services by promoting truly integrated care and the active involvement of users and carers in the planning and organisation of mental health services.

This article brought out the essential components of the implementation of an initiative that promotes users and carers’ involvement. This partnership is a form of normative integration which is designed to be flexible so it can be adapted to other local conditions and settings, based on the stakeholders involved [1]. In particular, the following characteristics of the CCIU should be emphasised: the small number of participants, the fact that at least half of the participants are users and carers, the absence of hierarchical relations between the participants, and direct contact between mental health workers, users and carers. Moreover, the following major elements need to be maintained: precise group rules that promote mutual respect and help to move beyond discussions on financial and power issues. The leadership of the CMHA-Montreal in coordinating and facilitating the CCIU also appears to be an essential component in the achievement of its goals. Several participants also referred to the importance of holding meetings outside their work settings so that a forum for dialogue and free speech can be created, supporting the emergence of a common language and fostering effective communication between the different stakeholders in the mental health network. The CCIU makes it possible to work on perceptions and attitudes and to develop a holistic view of services and shared values, an initial form of integrated care. This initiative also support users and carers implication in the mental health network by a better understanding of experiential knowledge contribution.

This logic analysis allowed the CMHA-Montreal to document the activities, mechanisms of actions and short-term and intermediate outcomes observed among the participants. More than that, the use of a strategic assessment approach allowed, as expected, the creation of a common understanding of the CCIU for the participants. The constructive process makes it possible for the participants to communicate, share their ideas and visions, and identify the impact of their participation in the group. A second phase of systematic evaluation of the effects of the CCIU experience is currently being conducted to document its outcomes in the participants’ practice settings and support the introduction of integrated care [24]. Some evaluation questions emerge of this logic modeling: How the CCIU has an impact on the development of a mutual trust? At which level participants reduce their prejudices and false perception? How many participants integrate in their organisation and practice an active involvement of users?

The CCIU’s future prospects are structured around the following main objectives: (1) documenting outcomes outside CCIU sessions in the different participants’ networks and; (2) setting up similar initiatives in Montreal and elsewhere in Quebec (Canada). Consequently, efforts are ongoing to disseminate the CCIU initiative among various integrated service networks in Quebec in order to promote its implementation in mental health networks (see Fleury [50] for the concept of integrated service networks). Implementation guides (tool kits) describing the resources needed and the components that are essential to its smooth operation have been developed and the model has been the subject of several conference presentations. A promotion committee has also been set up, composed of CCIU members, which represent a concrete outcome, in order to promote the approach among managers and clinicians in the various local mental health networks.

### Limitations

This analysis contains some limitations. First, despite several meetings with the CMHA-Montreal and the CCIU participants as well as participant observation carried out over the course of 1 year, additional meetings over a longer period of time would have allowed for the process to be deepened, in particular to identify long-term outcomes through qualitative and mixed methods approaches [4]. Moreover, although logic analysis was an essential step in the evaluation of this programme, it would be useful in the future to link it with a more systematic analysis of its real effects. Logic analysis helps, on one hand, to enhance the understanding of a programme and, on the other hand, to identify the mechanisms of action and outcomes. However, it does not make it possible to draw conclusions regarding its effectiveness.

## Conclusions

This logic analysis helped to better understand the mechanisms of action, the essential components and the outcomes of the CCIU. It allowed us to document a highly promising integrated care initiative that integrates users and carers in its forum for dialogue. This analysis enabled the CMHA-Montreal to develop tools to promote this initiative in the mental health networks and to pursue a programme evaluation process aimed at strengthening its outcomes. Evaluative participatory research is also one of the means used to co-construct an explanation, enable dialogue and foster cohesion among a group of stakeholders [51, 52]. Logic modelling of the CCIU helped to identify the key elements explaining the changes in perceptions and attitudes as well as the common vision of services acquired by its participants, thus contributing to practices centred on collaboration, partnership and quality care. Therefore, this approach constitutes an essential step leading to integrated care.

## Abbreviations

CCI: Carrefour Communautaire-Institutionnel (Connecting Community Organisations-Institutions)
CCIU: Carrefour Communautaire-Institutionnel-Usagers (Connecting Community Organisations-Institutions-Users)
CMHA-Montreal: Canadian Mental Health Association-Montreal Branch
